# CK19-positive Hepatocellular Carcinoma is a Characteristic Subtype

**DOI:** 10.7150/jca.44697

**Published:** 2020-06-28

**Authors:** Jian-Yong Zhuo, Di Lu, Win-Yen Tan, Shu-Sen Zheng, You-Qing Shen, Xiao Xu

**Affiliations:** 1 Department of Hepatobiliary and Pancreatic Surgery, the First Affiliated Hospital, Zhejiang University School of Medicine, Hangzhou, 310003, Zhejiang Province, China.; 2 NHC Key Laboratory of Combined Multi-organ Transplantation, Key Laboratory of the Diagnosis and Treatment of Organ Transplantation, CAMS, Hangzhou, 310003, Zhejiang Province, China.; 3 Department of Hepatobiliary and Pancreatic Surgery, Shulan (Hangzhou) Hospital, Hangzhou, 310003, Zhejiang Province, China.; 4 Center for Bionanoengineering and Key Laboratory of Biomass Chemical Engineering of Ministry of Education, College of Chemical and Biological Engineering, Zhejiang University, Hangzhou, 310003, Zhejiang Province, China.

**Keywords:** cytokeratin 19, hepatocellular carcinoma, subtype

## Abstract

The heterogeneity of hepatocellular carcinoma (HCC) commonly leads to therapeutic failure of HCC. Cytokeratin 19 (CK19) is well acknowledged as a biliary/progenitor cell marker and a marker of tumor stem cell. CK19-positive HCCs demonstrate aggressive behaviors and poor outcomes which including worse overall survival and early tumor recurrence after hepatectomy and liver transplantation. CK19-positive HCCs are resistant to chemotherapies as well as local treatment. This subset of HCC is thought to derive from liver progenitor cells and can be induced by extracellular stimulation such as hypoxia. Besides being a stemness marker, CK19 plays an important role in promoting malignant property of HCC. The regulatory network associated with CK19 expression has been summarized that extracellular stimulations which transmit into cytoplasm through signal transduction pathways (TGF-β, MAKP/JNK and MEK-ERK1/2), further induce important nuclear transcriptional factors (SALL4, AP1, SP1) to activate CK19 promoter. Novel noncoding RNAs are also involved in the regulation of CK19 expression. TGFβR1 becomes a therapeutic target for CK19-positive HCC. In conclusion, CK19 can be a potential biomarker for predicting poor prognosis after surgical and adjuvant therapies. CK19-pisitive HCCs exhibit distinctive molecular profiling, should be diagnosed and treated as a separate subtype of HCCs.

## Introduction

Hepatocellular carcinoma (HCC) emerges as a major health care problem globally. It is the fifth most common malignancy and the third cause of cancer-related death worldwide 1, 2. To date, surgical resection, liver transplantation, transcatheter arterial chemoembolization (TACE), radiofrequency ablation (RFA) and systemic therapies are the main treatment options to prolong life expectancy of patients with HCC 3. Unfortunately, 5-year overall survival rate is only 30-40% even after surgical resection and 70% of patients suffer from tumor recurrence within 5 years 3, 4. Besides that, the effectiveness of chemotherapy, molecular-targeted therapy or even immunotherapy to HCC is insubstantial 2, 5.

HCC is heterogeneous not only not only in terms of morphological characteristics and clinical behaviors, but also in genetic and molecular patterns 6. The inter-tumor and intra-tumor heterogeneity of HCC results in high resistance to therapeutic interventions 7, 8. Several molecular subtypes based on transcriptomic analysis have shown significant improvement in describing inter-tumor heterogeneity of HCC 9-12. As shown in previous studies, HCCs are divided into two groups according to their molecular features, namely, the proliferative and non-proliferative subtypes 11, 13, 14. Notably, cytokeratin 19 (CK19) is the vital marker of the proliferative subtype which indicates a poor prognosis in HCC patients 15.

Approximately 10-30% of HCC patients present CK19 expression 16-19. CK19-positive HCC is also known as biphenotypic HCC; that is, having the pathological features of both HCC and cholangiocarcinoma (CC). These group of patients often show worse outcomes as compared to the CK19-negative HCC patients. Therefore, inhibiting the incidence of CK19-positive HCC is of great significance.

As of now, the origin of CK19-positive HCCs, as well as the regulation of expression of CK19 in HCC, remains uncertain. In this review, we highlight recent advances in the overall perspective of CK19 in HCC and its relevant therapeutic implications.

## Pathophysiological change of CK19 in Liver

CK19 is an intermediate filament with a molecular weight of around 40 kDa. During the embryonic development, CK19 was detected in the primitive hepatic progenitor cells at the 4-10 weeks' gestation. Along with the development of fetal liver, these bipotential progenitor cells differentiate either into hepatocytes or biliary epithelial cells 20. However, the expression of CK19 is vanished in mature liver hepatocytes while it is constantly present in biliary epithelial cells. 20-22. Therefore, this explained the reason why CK19 became a significant marker of biliary epithelial cells in pathological diagnosis 23-25.

When hepatocytes suffer from damage in various chronic liver diseases such as chronic hepatitis, (non-) alcoholic steatohepatitis and haemochromatosis, the normal liver cells which are previously quiescent will be activated and transformed into progenitor cells 26-28. That is, CK19 re-emerges on hepatocytes anew when the resting cells are activated in the presence of inflammation or other stimuli. Meanwhile, a large number of studies showed that CK19 also appears aberrantly in HCC in which the incidence rate varied between 10 to 30% 16-19 (Table 1).

## CK19-positive HCC is associated with more aggressive behavior and poorer prognosis

CK19-positive HCC cells showed strong association with invasion, epithelial-mesenchymal transition (EMT) and angiogenesis. It became apparent when knockdown of CK19 successfully inhibited the invasive capacity and EMT in human HCC cells 19, 29, 30. CK19-positive cells are thought to be positively associated with angiogenesis as well 29. Kawai et al. 30 demonstrated that FACS-isolated single CK19-positive cells displayed high proliferation capacity and these cells are more resistant to chemotherapy such as doxorubicin and 5-fluorouracil 19, 30. A considerable number of researches have reported that CK19 is closely related to cancer stem cells which thus explained the resistance of CK19-positive cells to chemotherapy 30-32.

Patients with CK19-positive HCC have poorer prognosis compared with CK19-negative ones. As shown in Table 1, a substantial number of studies demonstrated that CK19 was associated with early tumor recurrence and worse overall survival after surgical resection or liver transplantation 18, 19, 21, 31. A meta-analysis enrolling 2943 HCC patients also demonstrated that CK19 overexpression was significantly associated with declined OS rate and 1-year DFS rate 33. After treated with TACE or RFA, CK19-positive HCC patients were also more inclined to early intrahepatic tumor recurrence 34, 35. Furthermore, vascular invasion and lower degree of differentiation were more commonly seen in CK19-positive HCC patients when compared with the negative counterpart 19, 21, 29. In order to comprehensively compare the difference of clinical parameters and physio-pathological features between CK19-negative and CK19-positive HCC CK19, we obtained accessible data from published literatures for analysis as shown in Figure 2 16, 17, 21, 29, 36-39.

CK19-positive HCC and CK19-negative HCC were compared with intrahepatic cholangiocarcinoma (ICC) and combined HCC and cholangiocarcinoma (cHCC-CC). The overall survival rate of CK19-positive HCC was similar with that of cHCC-CC where they were both significantly lower than the overall survival rate of CK19-negative HCC but higher than that in ICC 17. This indicated that the biological behavior of CK19-positive HCC was close to that of cHCC-CC.

Hoshida (S1-S3), Boyault (G1-G6) etc. have depicted several subtypes of HCC based on transcriptomics. As a matter of fact, CK19 is commonly described as a progenitor feature as it is closely associated with several stemness-related markers (EPCAM, NOTCH) 15, 40. Furthermore, CK19-positive subtype has been shown to have correlation with Hoshida S2, G1, proliferation subtype, Cluster A, iClust1 40, 41, and all these subtypes were related to poor differentiation, higher serum AFP level, higher frequency of vascular invasion and worse outcome 41. Taken together, CK19-positive HCC showed higher malignancy and worse outcome that it should be diagnosed and treated as a separate entity of HCCs.

## The origination of CK19-positive HCC

**Figure 1:**
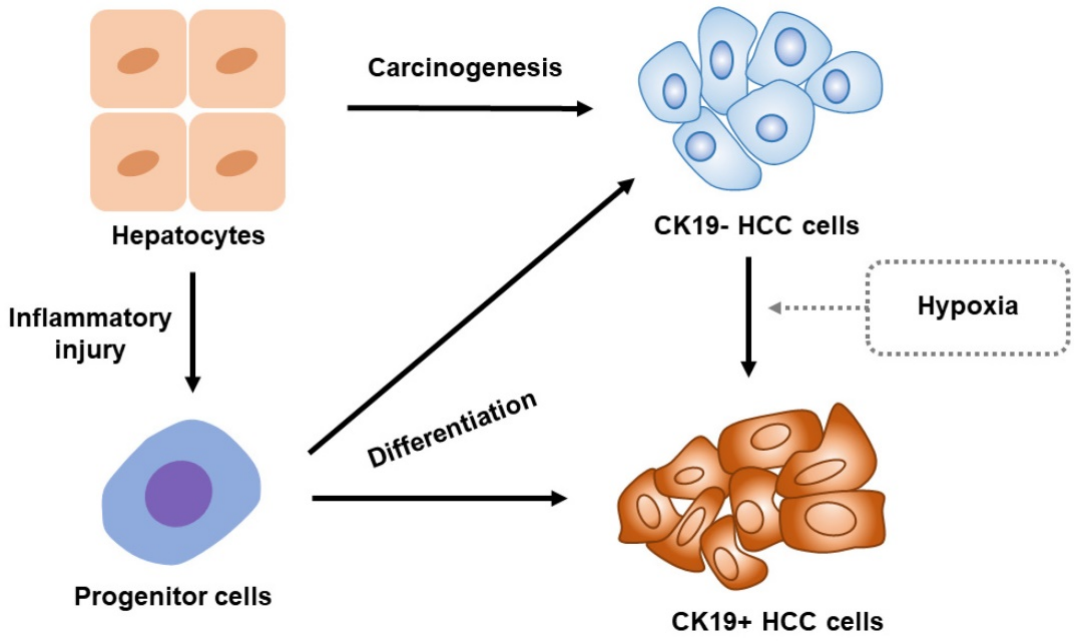
** Overview for the origin of CK19-positive HCC.** CK19 expression vanishes in adults' hepatocytes. Once the liver is damaged by inflammation, a portion of hepatocyte will reverse into progenitor cells which express CK19. These progenitor cells will potentially differentiate into CK19-positive HCC cells. Notably, the hepatocytes develop into CK19-negative HCC cells preferentially under normal condition. The hypoxia stimulations including the oxygen-deficient environment in tumor and hypoxic status caused by local treatments such as TACE will, however, induce CK19-negative HCC cells to transform into CK19-positive HCC cells. Abbreviation: CK19-, CK19-negative; CK19+, CK19-positive.

The molecular expressions of the HCCs are highly heterogeneous as they differ even within a single nodule of HCC. The heterogeneity of HCC is related to the origination of tumor cells which include hepatocyte or adult stem/progenitor cells. However, the origination of CK19-positive HCC remains controversial. Therefore, we illustrate an overview of CK19-positive HCC, as shown in Figure 1.

### CK19-positive HCCs originate from hepatocytes/liver progenitor cells

In normal liver, progenitor cell, one primordial component, is both positive for hepatocyte-differentiated (HEP-PAR) and biliary-differentiated (CK19) markers 18. At the time of liver tissue damage, either quiescent progenitor/stem cells are activated 42, or hepatocytes are dedifferentiated into immature progenitors or biliary-type cells 43, 44. Plentiful evidence suggested that these activated progenitor cells can be potential target cell during the development of HCC 45-47. Thus, the origination of CK19-positive HCC cells was presumed to be a cluster of progenitor cells.

Lee et al. 14 integrated the gene expression data from rat fetal hepatoblasts and adult hepatocytes with HCC from human and mouse models followed by subsequent classification of HCCs into two subtypes (HB and HC subtype). They demonstrated that HCC of the HB subtype arose from bipotential hepatic progenitor cells, and found that expression of CK19 was significantly higher in the HB subtype of HCC. This reflected that CK19-positive HCCs might be derived directly from liver progenitor cells.

Researchers from Columbia University 48 demonstrated that hepatocytes functioned as a cellular source for HCC and these liver progenitor cells found in HCC were derived from hepatocytes. This suggested that hepatocyte-derived HCC may dedifferentiate into a progenitor-like immature phenotype. As CK19 is considered as one of the progenitor cell markers, this may suggest that hepatocyte-derived HCC can dedifferentiate into CK19 positive HCC during the development of tumorigenesis.

If all HCC cells were originated from hepatocytes, these HCCs would only express hepatocyte-related markers (HEP-PAR, CK8 and CK18). As CK19 is increasingly regarded as a marker of bipotential hepatic progenitor cells, it is reasonable to hypothesize that CK19-positive HCCs were developed from hepatic progenitor cells.

### CK19-positive HCCs originate from environmental stimulation

The transformation of CK19-negative tumor cells into CK19-positive tumor cells is assumed to be an adaption to the specific challenges in the environment such as hypoxia and physical/chemical stimulation. Several studies have illustrated that carbonic anhydrase IX (CAIX), a hypoxia marker, was positively associated with CK19 after the TACE treatment 49, 50. Also, the incidence of CK19 positivity was significantly increased as the sessions of TACE increased 50. As shown in Table 3, the CK19 positive rate in the patients with TACE was higher than that in the non-TACE patients. These studies indicated that HCC with CK19-positive phenotype might originate from the transformations due to the anti-cancer and/or ischemic effects of TACE. Yoshida et al. 51 reported that the residual tumor due to incomplete RFA could be reactivated in the presence of thermal and hypoxic stimulation. These reactivated tumor cells usually portray higher malignancies and more aggressive invasive capabilities. In this study, HCC cell was exposed to high temperature which simulated the marginal zone of RFA treatment. *In vitro* cells survived from mimic-RFA showed an increase in CK19 expression.

Taken these evidences together, a new and inspiring hypothesis has been put forward that CK19 negative HCC may transform into CK19 positivity after patients receive several adjuvant therapies (e.g. TACE, RFA) as tumor environmental is stimulated. In addition, it provides a potential possibility for tumor progression and recurrence after treatment, and indicates a potential prevention strategy, that is, the CK19 regulatory network should be controlled in advance.

## Molecular characteristics of CK19-positive HCC

**Figure 2:**
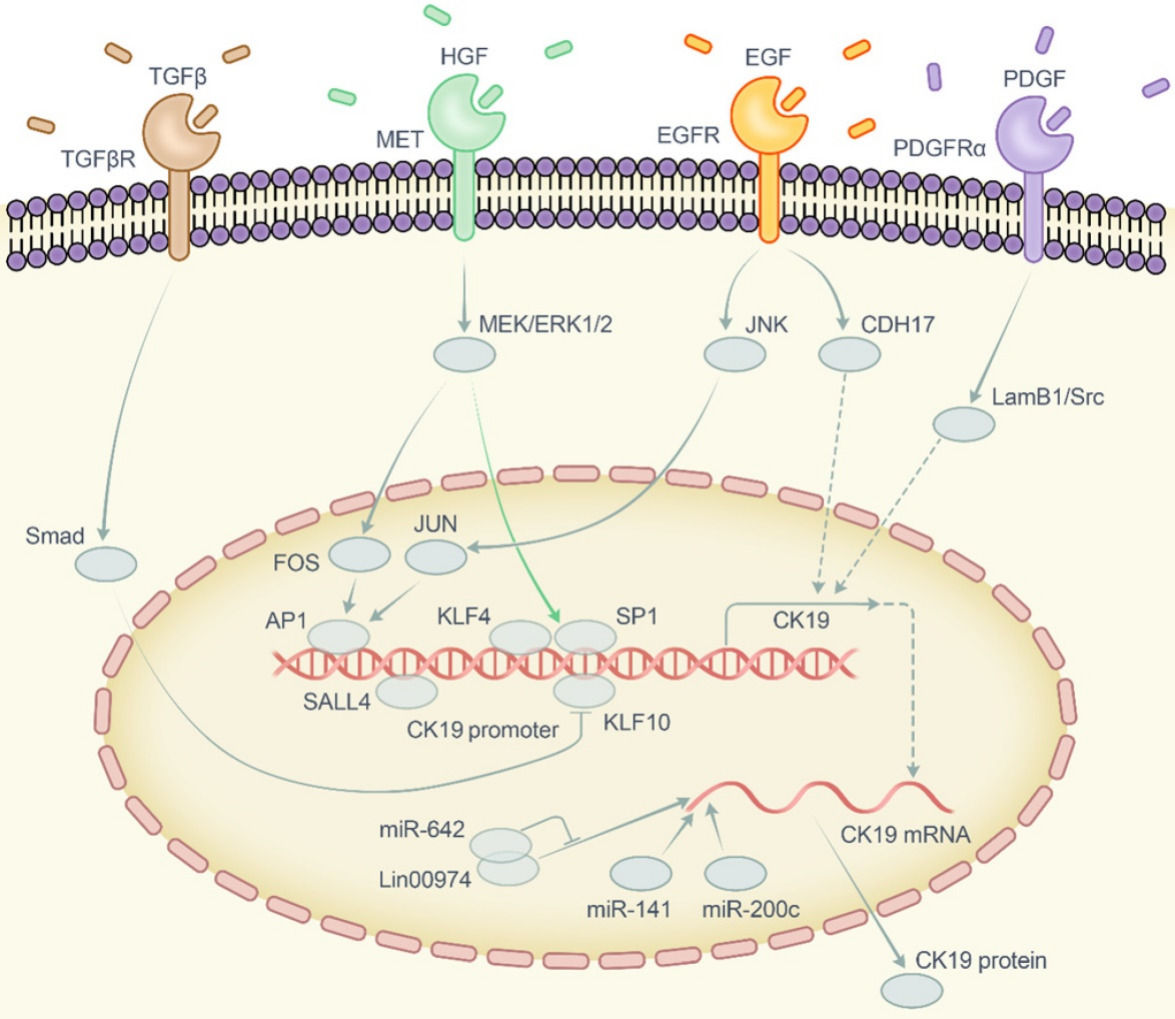
** Diagrammatic sketch of the regulatory network of CK19.** Abbreviation: CK19, cytokeratin 19; TGFβ, transforming growth factor beta; TGFβR, transforming growth factor beta receptor; HGF, hepatocyte growth factor; MET, MET proto-oncogene receptor tyrosine kinase or hepatocyte growth factor receptor; EGF, epidermal growth factor; EGFR, epidermal growth factor receptor; PDGF, platelet derived growth factor; PDGFRα, platelet derived growth factor receptor alpha; Smad, drosophila mothers against decapentaplegic protein; MEK, mitogen-activated protein kinase kinase 7; ERK, mitogen-activated protein kinase 1; JNK, mitogen-activated protein kinase 8; JUN, Jun proto-oncogene AP-1 transcription factor subunit; FOS, Fos proto-oncogene AP-1 transcription factor subunit; AP1, activator protein 1; SP1, specificity protein 1; KLF4, Krüppel like factor 4; KLF10, Krüppel like factor 10; SALL4, spalt-like transcription factor 4; CDH17, cadherin 17; LamB1, laminin subunit beta 1; Src, SRC proto-oncogene non-receptor tyrosine kinase; miR-642, microRNA 642a; miR-141, microRNA 141; miR-200c, microRNA 200c; Lin00974, long intergenic non-protein coding RNA 974.

Despite the clinical significance of CK19 in HCC, the role of CK19 in tumor is still ambiguous in which whether it functions as a phenotype marker or an oncogenic factor has yet to be speculated. Over the past two decades, CK19-positive cells have gradually been regarded as a kind of stem cell 52 as well as an important prognostic marker of HCC as described above. Recent studies have also showed that CK19 enhanced the tumorous properties in breast cancer, colon cancer and hepatocellular carcinoma 19, 53, 54. These data implied that CK19 plays an important part in carcinogenesis. However, the molecular network of this phenotype is not unraveled completely. Therefore, we summarized and highlighted the vital pathways involved in regulating the CK19 expression which include the carcinogenic growth factors and corresponding receptors, MAKP/JNK and MEK-ERK1/2 pathways, transcription factors and noncoding RNAs (Figure 2).

### Extracellular stimulation: Carcinogenic growth factors

Belgian researchers 19 previously revealed that CK19 was associated with platelet-derived growth factor receptor α (PDGFRα). Furthermore, they demonstrated that PDGF could elevate CK19 expression via PDGFRα-La/SSB-LAMB1 axis 55. A study from Kanazawa University 21 showed that epidermal growth factor (EGF) had potent effects on promoting CK19 expression *in vitro*. Another Korean study showed that hepatocyte growth factor (HGF) from cancer-associated fibroblasts (CAF) could upregulate CK19 expression based on cross-talk between CAF and HCC cells 56. Apparently, the transforming growth factor-β (TGF-β) is another important extracellular factor involved in the progressive features of CK19-positive HCC cells as the inhibition of transforming growth factor-β receptor 1 (TGFβR1) could significantly attenuate the proliferation capability of CK19-positive HCC 30. Albeit no obvious evidence supporting the direct regulation between TGFβ and CK19, TGFβ/Smad signaling exhibits enormous association with CK19-positive HCC as described above. This collectively indicates that these carcinogenic growth factors with corresponding receptors engage in regulating the CK19 expression.

### Signal transduction: JNK, MEK-ERK1/2 and Smads signaling pathway

Yoneda et al. 21 demonstrated that c-Jun-N-terminal kinase (JNK)/stress-activated protein kinase (SAPK) is a downstream signaling pathway involved in regulating CK19 expression via EGF-EGFR. In another study, HCC tumor specimens with matched distal noncancerous liver tissue were divided into two subgroupes according to JNK1 activation status. As a result, CK19 was over expressed in high JNK1 HCC 57. American investigators 58 evaluated the transcriptomic differences between CK19-positive and CK19-negative foci through the resistant hepatocyte (RH) rat models to select unique genes in each group. The connectivity of the top regulatory networks showed a dominant enrichment of AP-1/JUN in CK19-positive areas. These data suggested a hypothesis that JNK pathway is involved in regulating CK19 expression.

MEK-ERK1/2 pathway is another vital intracellular signaling pathway participated in the modulation of CK19 expression. Rhee et al. proposed that, MET, the receptor of extracellular signal HGF, upregulated CK19 expression via activating MEK-ERK1/2 pathway 56. MET is also known to be related to poor prognosis and HGF/MET signaling axis currently became an emerging therapeutic target of HCC 59. Therefore, CK19-positive HCCs are deemed to be accompanied with the activation of HGF/MET signaling. In Kawai's study, TGFβ/Smad signaling is activated in CK19-positive cells 30 and it is always reasonable to assume that Smad pathway is part of the regulatory network of CK19 expression.

### Endonuclear activation: Nuclear transcription factors

Regulatory pathways relay the signal to transcription factors and further activate the CK19 promoter. Two important transcription factors, namely activator protein 1 (AP1) and specificity protein 1 (SP1), have been identified as regulators that bind directly to the CK19 promoter site 56. Besides that, AP1 and SP1 are also known as the downstream transcriptional activators of ERK1/2. JUN and FOS proteins which belong to the AP1 family can be dimerized to form JUN homodimers or JUN/FOS heterodimers in order to become valid transcriptional factors 60. As mentioned above, JNK pathway is also involved in regulating CK19 expression via EGF-EGFR while FOSL1, a protein of FOS family, can be activated by ERK1/2 pathway 56. In short, we conclude that AP1 serves as the downstream transcriptional factor of both JNK and ERK pathway for CK19 regulation.

Recently, overexpression of Sal-like 4 (Drosophila) (SALL4) was reported to upregulate CK19 expression at both the mRNA and the protein level 61. SALL4 is an important transcription factor in HCC which correlates to stemness, 5-FU resistance and differentiation 62. We suggest that SALL4 might bind to the promoter site of CK19.

Two members from Krüppel-like Factors (KLF) family, which are also DNA-binding transcriptional regulators, have shown an association with CK19. Brembeck from University of Pennsylvania verified that the CK19 gene is regulated by the interplay of Krüppel-like factor 4 (KLF4) and SP-1 through a critical cis-regulatory element in the proximal promoter 63. On the other hand, Andersen et al. 58 examined the functional connectivity among the significant genes and found that the most predominant feature in the CK19 negative focal lesions was the overexpression of Krüppel-like factor 10 (KLF10) in rat model. As murine CK19 gene exhibits high homology to human counterpart 64, we can translate these findings to human being. Besides, KLF10 has been validated as a tumor suppressor gene and shown association with TGFβ/Smad signaling pathway 65. Therefore, we hypothesize that KLF10 may inhibit CK19 promoter activity by suppressing TGFβ/Smad signaling. Above all, transcription factors such as AP-1, SP-1, SALL4 and KLF family (KLF4, KLF10) might regulate the expression of CK19 either directly or indirectly by interacting with its promoter binding site.

### Non-coding RNA and others

Tang et al. 66 showed that long noncoding RNA (lncRNA), Linc00974, presented positive regulation of CK19 via posttranscriptional modification. It functions as a sponge to endogenously compete with the suppressive effect of miR-642 to CK19. Moreover, miR-200 family (miR-141/miR-200c) especially miR-141 was identified to have strong linkage with CK19 expression 19. That is, overexpression of miR-141 and miR-200c significantly upregulated the expression of CK19 in CK19-negative or CK19-low HCC cells. Lee et al. 67 detected 20 patients with hepatitis B virus (HBV)-HCC through Affymetrix U133A oligonucleotide microarray. It was shown that cadherin 17 (CDH17) was positively correlated with CK19 expression and CDH17 could enhance the expression of CK19 through EGF/EGFR-CDH17-CK19 pathway.

Make a brief summary, as CK19 has been regarded as a marker of progenitor- and stem-cells, previous publications have done a body of studies to clarify how to regulate the expression of CK19 as shown in Figure 2. The internal regulatory network, however, is more complex and intricate than what we have seen so far. Govaere et al. also reported that CK19 showed a robust positive correlation with other 'aggressive' phenotypes and signatures including 'poor survival HCC subtype', 'proliferation HCC subtype', 'S1 signature with aberrant Wnt activation subtype' and so on 19. Therefore, attention should not be focused solely on CK19 as a target that function as a biomarker and oncogenic factor. CK19-positive HCC should be regarded as a completely different phenotype and an independent entity with its own characteristics.

## The detective and therapeutic strategy for CK19 positive HCC

### Non-invasive detection method for CK19 expression

The current detection accesses of CK19 are mostly dependent on the post-operative immunochemistry. Advanced acquisition of CK19 expression level can guide clinical physicians to choose optimal therapeutic methods before surgery or locoregional treatment. CYFRA 21-1 is a soluble fragment of CK19 in peripheral circulation. In fact, serum CYFRA 21‐1 has been detected in various malignancies including non‐small cell lung cancer, esophageal cancer, breast cancer and pancreatic cancer 68-71. Therefore, serum CYFRA 21‐1 is considered as a useful biomarker to indicate CK19 expression in HCC 72.

More recently, radiological examination has become a novel non-invasive diagnostic method for CK19-positive HCC. Choi et al. 73 determined the preoperative magnetic resonance (MR) imaging characteristics of HCC potentially related to CK19 expression. Another study illustrated that MR features combining with elevated AFP were able to distinguish CK19-positive HCCs from CK19-negative HCC 74. Therefore, application of these non-invasive detection methods will be conducive to monitor the CK19 expression status.

### The therapeutic strategy for CK19-positive HCC

Considering the different molecular features and the distinct invasive properties of CK19-positive HCCs, this subtype of HCCs should be regarded as a separate entity which is different from the CK19-negative HCCs 19. We would like to regard the presence of CK19 as a therapeutic-phenotype to guide individualized treatment in clinical context as it is of utmost important to deliberate the strategies thoroughly to overcome the highly tortuous CK19-positive HCCs.

As aforementioned, Japanese researchers discovered that TGFb/Smad signaling was activated in CK19-positive HCC cells. In other words, TGFβR1 inhibitor (LY2157299) could effectively inhibit the proliferation of CK19-positive HCC 30. Therefore, TGFbR1 inhibitor should be considered as a new targeted-therapy against CK19-positive HCC.

A Chinese study enrolling 280 HCC patients who were either treated with or without sorafenib after surgical resection showed no difference between two groups in terms of overall survival. Intriguingly, patients who have received sorafenib treatment after surgery exhibited superior overall survival compared to those who have not received sorafenib treatment after surgery in CK19^+^OV6^+^ subgroup 75. This implied that patients with CK19-positive HCC could benefit from sorafenib administration after surgery. As Govaere et al. 55 demonstrated that CK19 could be progressed through PDGFRα-LAMB1-CK19 axis, inhibitors of PDGFRα such as imatinib 76, regorafenib 77 and lenvatinib 78 might exert specific effect on CK19-positive HCC. Therefore, the above-named first-line or second-line multi-targeted tyrosine kinase inhibitors (TKI) approved by FDA for HCC are supposed to have therapeutic priority to CK19-positive HCC. There is no doubt that more clinical trials are needed to validate the most efficacious drug in dealing with CK19-positive HCC.

## Conclusions

CK19-positive HCC demonstrates more aggressive behaviors and poorer prognosis. The complex internal regulatory network makes CK19-positive HCC an independent entity with its own characteristics, which should be diagnosed and treated as an independent subtype. Clarification of the internal molecular mechanisms is urgently needed to prevail over CK19-positive HCCs in the clinical management.

## Figures and Tables

**Table 1:** Clinical prognosis comparison between CK19-negative and CK9-positive HCC

Author	Year	Source of specimen	Cases of CK19+	Prognosis-related indexes		Note
				CK19-	Ck19+	
Wu et al. 18	1996	Needle biopsy/ surgical resection/ autopsy	79 (27.24%)	30.7%	17.6%	1.5y OS
Lee et al. 17	2012	Surgical resection	21 (30.00%)	80.4%	28.9%	5y OS
Fatourou et al. 38	2015	Surgical resection/ liver transplantation	9 (10.11%)	56%	15%	5y OS $
Miltiadous et al. 40	2015	Liver transplantation	58 (43.94%) #	67%	44.9%	5y OS
Takano et al. 29	2016	Surgical resection	12 (8.82%)	90%	61%	5y OS $
Lee et al. 37	2017	Liver transplantation	8 (36.36%)	82.3%	55.4%	5y OS
Yang et al. 36	2008	Surgical resection	45 (18.75%)	57.2%	37.8%	7y OS
Yoneda et al 21	2011	Surgical resection	9 (11.54%)	64%	28%	2y DFS $
Uenishi et al 39	2003	Surgical resection	15 (9.55%)	44%	20%	3y DFS
Durnez et al. 16	2006	Surgical resection/ needle biopsy/ liver transplantation	18 (16.51%)	95%	50%	3y RFS $
Lee et al. 17	2012	Surgical resection	21 (30.00%)	54.5%	34.3%	3y DFS

Abbreviation: HCC, hepatocellular carcinoma; CK19, cytokeratin 19; OS, overall survival; DFS, disease-free survival; RFS, recurrence free survival; CK19-, CK19-negative; CK19+, CK19-positive. #, CK19/S2 + gene expression signature. $, the data of overall survival (OS) and disease-free survival (DFS) were measured by computer image-scale comparison.

**Table 2:** The clinical parameters and physio-pathological features compared between CK-negative and CK19-positive HCC

		CK19-	CK19+	P value
Gender				**0.007**
	Male	650 (81.4)	100 (71.4)	
	Female	149 (18.6)	40 (28.6)	
Cirrhosis				0.651
	Yes	414 (58.6)	76 (60.8)	
	No	292 (41.4)	49 (39.2)	
HBV				**0.010**
	Yes	331 (43.2)	76 (55.1)	
	No	435 (56.8)	62 (44.9)	
HCV				**0.003**
	Yes	269 (35.0)	30 (21.9)	
	No	499 (65.0)	107 (78.1)	
Preoperative AFP (ng/ml)				**0.047**
	≤20	221 (49.6)	30 (37.5)	
	>20	225 (50.4)	50 (62.5)	
Tumor number				0.796
	Single	509 (78.9)	95 (77.9)	
	Multiple	136 (21.1)	27 (22.1)	
Tumor size (cm)				0.545
	>5	200 (43.1)	41 (46.6)	
	≤5	264 (56.9)	47 (53.4)	
Differentiation				**<0.001**
	Well/moderate	338 (71.2)	34 (45.3)	
	Poor	137 (28.8)	41 (54.7)	
TNM stage				0.575
	I-II	399 (67.9)	67 (65.0)	
	III-IV	189 (32.1)	36 (35.0)	
Microvascular invasion				0.099
	Yes	128 (54.5)	28 (68.3)	
	No	107 (45.5)	13 (31.7)	
Portal vein invasion				**0.033**
	Yes	89 (35.0)	16 (55.2)	
	No	165 (65.0)	13 (44.8)	
Metastasis				0.585
	Yes	52 (11.1)	12 (13.0)	
	No	418 (88.9)	80 (87.0)	

Abbreviation: HCC, hepatocellular carcinoma; CK19, cytokeratin 19; CK19-, CK19-negative; CK19+, CK19-positive. Note: The data were obtained from published literatures 16, 17, 21, 29, 36-39. The parameters were analyzed using Chi square test. P <0.05 was considered as significantly different.

**Table 3:** The comparison of CK19 positive rate in HCC patients receiving TACE treatment before operation

Author	Year	Treatment	Total cases	Treat group		Non-treat group	
				Ck19-	Ck19+	Ck19-	Ck19+
Lai et al. 49	2015	TACE	57	34	6	17	0
Nishihara et al. 35	2008	TACE*	226	61	19	128	18
Zen et al 79	2011	TACE*	80	32	8	40	0
Rhee et al. 50	2016	TACE*	85	38	13	32	2

Abbreviations: HCC, hepatocellular carcinoma; CK19, cytokeratin 19; TACE: transcatheter arterial chemoembolization; CK19-, CK19-negative; CK19+, CK19-positive. *, p<0.05, Fisher's Exact Test.

## Abbreviations

HCC: hepatocellular carcinoma
CK19: cytokeratin 19
TACE: transcatheter arterial chemoembolization
RFA: radiofrequency ablation
TGFβ: transforming growth factor beta
TGFβR: transforming growth factor beta receptor
HGF: hepatocyte growth factor
MET: MET proto-oncogene receptor tyrosine kinase or hepatocyte growth factor receptor
EGF: epidermal growth factor
EGFR: epidermal growth factor receptor
PDGF: platelet derived growth factor
PDGFRα: platelet derived growth factor receptor alpha
Smad: drosophila mothers against decapentaplegic protein
MEK: mitogen-activated protein kinase kinase 7
ERK: mitogen-activated protein kinase 1
JNK: mitogen-activated protein kinase 8
JUN: Jun proto-oncogene AP-1 transcription factor subunit
FOS: Fos proto-oncogene AP-1 transcription factor subunit
AP1: activator protein 1
SP1: specificity protein 1
KLF4: Krüppel like factor 4
KLF10: Krüppel like factor 10
SALL4: spalt-like transcription factor 4
CDH17: cadherin 17
LamB1: laminin subunit beta 1
Src: SRC proto-oncogene non-receptor tyrosine kinase
miR-642: microRNA 642a
miR-141: microRNA 141
miR-200c: microRNA 200c
Lin00974: long intergenic non-protein coding RNA 974
